# Overview of Popular Techniques of Raman Spectroscopy and Their Potential in the Study of Plant Tissues

**DOI:** 10.3390/molecules26061537

**Published:** 2021-03-11

**Authors:** Aneta Saletnik, Bogdan Saletnik, Czesław Puchalski

**Affiliations:** Department of Bioenergetics, Food Analysis and Microbiology, Institute of Food Technology and Nutrition, College of Natural Sciences, University of Rzeszów, Ćwiklińskiej 2D, 35-601 Rzeszów, Poland; a.saletnik@ur.edu.pl (A.S.); cpuchal@ur.edu.pl (C.P.)

**Keywords:** Raman spectroscopy, spontaneous Raman scattering, stimulated Raman scattering, surface-enhanced Raman scattering (SERS), tip-enhanced Raman scattering (TERS), confocal Raman microscopy, chemical imaging

## Abstract

Raman spectroscopy is one of the main analytical techniques used in optical metrology. It is a vibration, marker-free technique that provides insight into the structure and composition of tissues and cells at the molecular level. Raman spectroscopy is an outstanding material identification technique. It provides spatial information of vibrations from complex biological samples which renders it a very accurate tool for the analysis of highly complex plant tissues. Raman spectra can be used as a fingerprint tool for a very wide range of compounds. Raman spectroscopy enables all the polymers that build the cell walls of plants to be tracked simultaneously; it facilitates the analysis of both the molecular composition and the molecular structure of cell walls. Due to its high sensitivity to even minute structural changes, this method is used for comparative tests. The introduction of new and improved Raman techniques by scientists as well as the constant technological development of the apparatus has resulted in an increased importance of Raman spectroscopy in the discovery and defining of tissues and the processes taking place in them.

## 1. Introduction

Biological material is characterized by a complex structure and varied chemical composition. Often the molecules tested are found in the sample at very low concentrations [1]. Instrument-based methods that can be used in the analysis of cell wall structure in plants are UV-VIS, fluorescence, infrared, Raman spectroscopy, and nuclear magnetic resonance spectroscopy [2]. Spectroscopic methods are analytical techniques in which a spectrum is generated as a result of the interaction of electromagnetic radiation with matter. They include, among other techniques, molecular spectroscopy, which deals with the interaction of electromagnetic radiation with molecules resulting in complex changes in the energy states of the molecules. As a result of absorption or emission of a quantum of electromagnetic radiation, transitions between the existing energy levels of atoms and molecules take place.

As a result of the absorption of electromagnetic radiation by the molecule, the necessary energy levels are excited. This will result in changes in the spectrum of the excitation radiation (absorption spectrometry) or an emission spectrum is generated due to the return of the molecule to the ground state. The distinction is made between the spectrum associated with the change of rotation, the vibration and the excitation levels of electrons. Raman spectroscopy examines the rotational and oscillatory-rotational spectra of molecules [3,4,5,6,7]. Raman light scattering was first observed in 1928 and was used to explore the vibrational state of molecules in the 1930s [5,6,7]. Raman applications on plants began in the 1984 with the investigation of cellulose. Raman spectroscopy has undergone a renaissance in the past decade [8].

## 2. Principle, Instrumentation

Raman spectroscopy is used to study the structure, dynamics of changes, and functions of biomolecules. In combination with microscopy, data with high spatial resolution is acquired [8]. Raman spectroscopy is a vibrational technique that does not utilize markers. It thus enables an insight into the structure of tissues and cells and permits an investigation of their composition at the molecular level thus playing a very important role in biological research [9,10]. Raman spectroscopy is designed to measure the frequency shift of inelastic scattered light when a photon of incident light hits a particle and produces a scattered photon [11,12,13,14,15,16]. A Raman spectrum consist of bands which are caused by an inelastic scattering from the chemically bonded structures [17]. The obtained Raman spectrum is composed of bands whose position depends on the vibration frequency of the sample components. Each organic compound and functional groups have a characteristic vibration frequency visualized in the Raman spectrum in the form of a peak [6]. The characteristic spectral pattern is the so-called fingerprint, owing to which we can identify the compound, while the intensity of the bands can be used to calculate its concentration in the analyzed sample [8].

**Figure 1:**
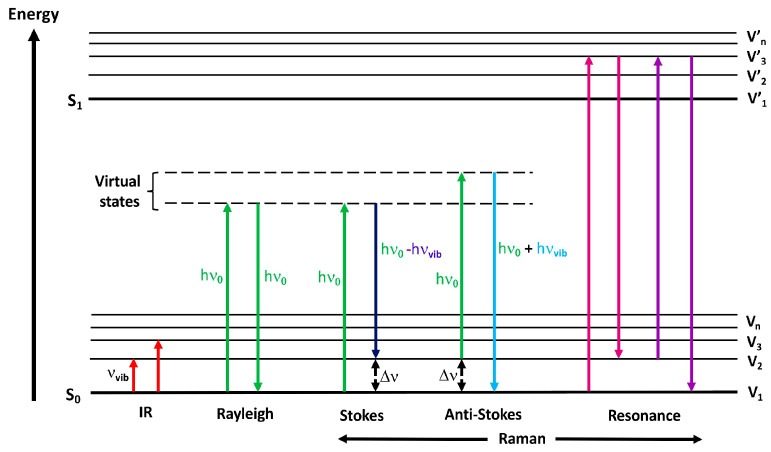
Jablonski energy diagram showing the transitions involved during infrared absorption, Rayleigh, Raman Stokes, anti-Stokes and Resonance Raman scattering.

In the light scattered by the test medium there is, for the most part, a component of the same frequency as in the incident light (Rayleigh scattering, elastic scattering) [18,19], while in a minority of cases there are variable frequency components associated with the change in photon energy (inelastic scattering, Raman scattering). The outgoing scattered light can be a photon with a frequency lower than the incident photon and in such cases we call it Stokes Raman scattering, or it is of a frequency that is higher, and then it is known as anti-Stokes Raman scattering [20]. The Stokes band forms when the molecule, after interacting with the excitation radiation, shifts to a higher vibratory level and the scattered photon has energy lower by the energy difference between the levels of vibrational energy. On the other hand, the anti-Stokes band may appear if the molecule was at the excited oscillatory level before the impact of the excitation radiation—that way there is a high probability that it will return to the basic oscillatory level. The scattered photon will have an energy greater by the difference in energy of the oscillating energy levels [21]. In anti-Stokes Raman scattering, the photon will collect energy from the bond of the molecule when the bond is initially in an excited vibrational state [22,23]. The diagram in Figure 1 shows schematically the Raman and Rayleigh scattering process.

The intensity of the anti-Stokes band is lower than that of the Stokes band because there are a very few molecules at the excited oscillatory level at room temperature. The intensity of the Raman scattering is approximately 106 times lower than the excitation radiation intensity. A strong source of electromagnetic radiation is used to excite the Raman spectrum. The excitation radiation usually comes in the form of visible light, but ultraviolet or near-infrared radiation can also be used. The selection of the optimal wavelength of excitation is related to the spectral characteristics of the tested particles. The magnitude of the shift of Raman bands relative to the Rayleigh bands does not depend on the frequency of the excitation radiation but on the properties of the scattering particles. Since the Stokes bands are of higher intensity than the anti-Stokes bands, in Raman spectroscopy the measurement most often concerns only the Stokes part of the Raman spectrum [21].

For the vibration to be visible, the Raman spectrum must meet the condition of changing polarizability during normal vibration of the molecule; the so-called selection rule in the Raman spectrum. According to the Raman selection rule, the scattering intensity is proportional to the magnitude of the change in molecular polarization. The change in the polarizability of molecules will result from the displacement of electrons from the equilibrium position as a result of molecular vibrations [24]. The intensity of the bands is proportional to the intensity of the excitation beam, the reciprocal of the fourth power of its wavelength, and the size of the polarization tensor of the corresponding vibration [25]. The greater the polarizability of a molecule, the less bound are the electrons in the atom [26]. The change of wavelength of the scattered light depends on the chemical composition of the structures responsible for its scattering [24].

Raman light scattering by molecules was first predicted by the classical Smekal quantum theory in 1923, and first experimentally observed by Sir Chandrasekhara Venkat Raman and his student Kariamanickam Srinivas Krishnan in 1928 [27,28]. In honor of the discoverer, the phenomenon of inelastic light scattering is called Raman scattering [29]. In 1930, Chandrasekhara Raman was awarded the Nobel Prize for his experimental confirmation of quantum-mechanical scattering theory [21].

Fifty years after the first observation, Raman spectroscopy became one of the main analytical techniques among techniques of optical metrology, especially when water and other polar solvents that absorb light in the infrared area are present in the sample [10]. In biological applications, Raman spectroscopy has the advantage that spectra containing a large amount of information can be obtained from intact tissue [24,30], so without interfering with its structure. Therefore, a detailed chemical analysis of biological material is possible, despite its high complexity. The Raman spectrum can be used as a fingerprint tool for various compounds [23]. Thus, the acquired spectrum of the analyte can be utilized as a qualitative analysis for unknown samples or a mixture of constituents [31]. Moreover, Raman spectroscopy is sensitive to even small structural changes, therefore comparative studies utilizing this method are carried out [30].

Raman scattering in tissues provides an abundance of information on the vibrational structure of their constituent proteins, GAGs, lipids, and DNA. Raman spectra are often recorded in the so-called fingerprint region (400–1800 cm^−1^), which contains relatively weak but highly specific Raman peaks. Recently, additional attention has been devoted to the use of a high wavenumber region (2800–3600 cm^−1^) which contains Raman bands that are less specific but show a higher degree of signal intensity [31]. An important advantage of Raman spectroscopy is also the low intensity of the water bands which render the analysis of biological materials very difficult in the infrared [29].

Despite a number of advantages, Raman spectroscopy also has disadvantages, especially in relation to biological samples. Using laser waves in the visible light range often causes excitation of fluorescence. Excitation in the absorption band can lead to interference of the Raman signal with fluorescence, and also, due to the high intensity of excitation radiation, cause sample decomposition [29].

The Raman spectrometer consists of a light source—a laser, a monochromator, a sample holder, and a detector. Lasers are currently used as excitation light sources in Raman spectroscopy. The lasers used may differ depending on the specificity of the research conducted. The choice of wavelength for laser excitation is one of the most important parameters when performing Raman spectroscopy. Several types of laser which emit light of a specific wavelength can be used as the excitation source: krypton (530.9 and 647.1 nm), helium-neon (632.8 nm), neodymium (1064 nm and 532 nm), argon (488.0 and 514.5 nm), and diode lasers (630 and 780 nm). The use of a 1064 nm near-infrared (NIR) excitation laser as a light source results in a weaker fluorescence effect than lasers with visible wavelength [32]. In general, wavelengths are selected based on a compromise between reducing background autofluorescence and maximizing the intensity of the Raman signal, both of which increase with decreasing wavelength (Raman signal scale with the fourth power of the excitation frequency). The optimization of this balance is highly dependent on the sample type and application. Most tissues give a sufficient Raman signal and the desired low autofluorescence as a result of near-infrared (NIR) laser excitation at 785–1048 nm [33,34]. However, there are exceptions as some tissues may show unacceptably high autofluorescence even with NIR excitation [35]. For samples with particularly low rates of autofluorescence, it is often advantageous to use shorter excitation wavelengths, such as 633 nm or 532 nm, which significantly speed up the acquisition time due to the generation of stronger signals. However, it should be noted that Raman spectra obtained at different wavelengths of laser excitation should not be directly compared due to the possible effects of molecular resonance. Excitation radiation is directed through a system of mirrors and lenses onto the test sample, where it is scattered. The imaging resolution of Raman microscopy depends on the specific type of system, but is ideally limited by diffraction and therefore highly dependent on the excitation wavelength and the selection of lens [31].

Over many decades, the use of Raman spectroscopy was limited due to the very low efficiency of normal Raman scattering, and the cost of parts in a spectrometer, which were additionally unsuitable for on-site analysis [23]. The development and refinement of Raman spectroscopy equipment allowed these limitations to be overcome and enabled spectroscopy to be used in a wide area of research. In recent decades, the technique of Raman spectroscopy has overcome the problems of fluorescence, poor sensitivity, or a weak Raman signal. To satisfy the ever-increasing demands of analysis, many advanced Raman techniques have been developed [18]. Currently, there are over 25 different types of Raman spectroscopy technique. For example, a Fourier transformation (FT) Raman spectrometer using a near-infrared (NIR) laser solves the problem of fluorescence interference [36]. The surface-enhanced Raman spectroscopy technique enhances the Raman scattering signal [37]. Raman confocal microscopy provides three-dimensional images of the structure and composition of the material with micrometric resolution and clear image quality [38]. Coherent Anti-Stokes Raman Scattering (CARS) provides spectral information with excellent sensitivity and low laser power [39]. Raman resonance scattering (RRS) allows the study of a spectrum of materials in the range of the photon energy itself [40].

Portable and relatively cheap hand-held Raman spectrometers are also now available [30]. The constantly improved portable Raman technology is more and more frequently used as a convenient, sensitive, and non-invasive tool for on-site inspection and evaluation of fruit and vegetables on croplands, in storage or in shops. Nekvapil et al. 2016 used a hand-held Raman spectrometer to test the freshness of citrus fruits widely available in the market. An analysis was made of the quantity of carotenoids in the peel selected species. Scientists have proven that citrus freshness can be assessed using a portable Raman spectrometer. The intensity of the carotenoid signal in a portable device has become a determinant of a new fruit freshness factor, the so-called Raman freshness coefficient (C_Fresh_), (CFresh), the course of which decreases with time to a varying extent for different citrus groups [41]. Feng et al. 2015 showed that a portable Raman spectrometer can be successfully used in cooking oil suitability tests. Analysis with a hand-held Raman spectrometer successfully detects impurities in the oils that are tested [42].

**Figure 2:**
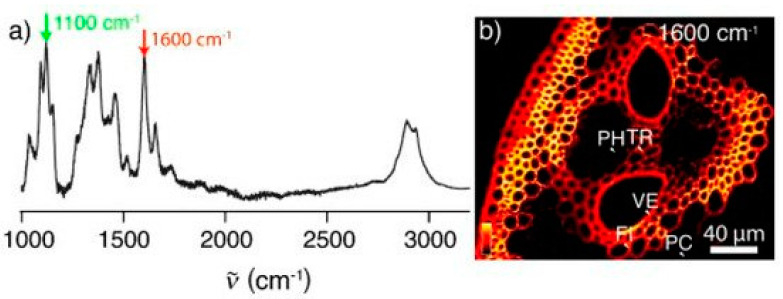
Raman spectrum of corn stover (**a**) and stimulated Raman scattering (SRS) microscopy imaging of corn stover at 1600 cm^−1^ (**b**) illustrating the lignin distribution.

Raman microspectroscopy provides spatial information on vibrations from complex biological samples, making it a very precise tool for the study of various plant materials [43]: pollen [44,45], fruit [46,47], roots [48], and wood of various origins [49,50,51,52,53,54]. Both the molecular composition and the molecular structure of the cell walls can be examined in the samples (example below Figure 2) [8,51,52,53,54,55,56,57].

This overview provides a practical introduction to the science of Raman spectroscopy. Selected Raman techniques and their use in various fields of science will be briefly described. Particular attention is paid to the method of confocal Raman spectroscopy used in research on the analysis of plant cell walls. The latest results of Raman research, based on the findings, are summarized illustrating the current and potential future applications of Raman spectroscopy in plant cell wall studies.

## 3. An Overview of Selected Raman Techniques

### 3.1. Surface-Enhanced Raman Spectroscopy (SERS)

**Figure 3:**
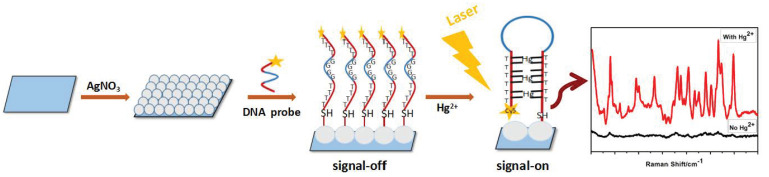
Mechanism of surface-enhanced Raman scattering sensor (SERS) for the detection of mercury ions (Hg^2+^) using silver colloids.

SERS is one of the most sensitive devices, allowing the detection of very low concentration analytes [58]. The SERS technique is based on the amplification of the scattered Raman signal by the particles absorbed on the metal surface. The signal amplification is the effect of the interaction of electromagnetic waves with metal in the phenomenon known as plasmon resonance [59,60]. SERS is characterized by a large increase in the cross-section for the Raman scattering of the analyte, even up to 15 orders of magnitude, compared to the conventional Raman method [23,61]. In the SERS method, the gain in signal intensity can even be 10^6^ times greater than with classical Raman spectroscopy. To achieve such a high gain, the particles should be absorbed on the surface of the metal substrate or be very close to it (approximately 10 nm). The change in the intensity of the output signal is influenced by the type of metal, its roughness, size and shape, and the intensity of the incident light [59,60]. Colloidal metals and rough electrodes are used as active surfaces in SERS. Due to their morphology and size, colloidal metals are easy to prepare and describe [62]. The first recorded SERS spectra were made using a roughened silver electrode [63]. Gold and copper were subsequently tested as metal surfaces. All these metal surfaces increased the intensity of the Raman signal 104 to 106-fold. Due to their properties, the most commonly used types of the substrate are silver and gold, as they are the most stable in air [58]. Even though gold systems have lower EMF intensification factors than silver systems in some analyses, they are a more suitable plasmonic material than silver. Due to its high chemical stability and biocompatibility, gold is more often selected for testing biological samples in the SERS method [64,65]. Figure 3 shows an example of the use of the surface enhanced Raman scattering (SERS) method to detect mercury (Hg^2+^) ions in a diagram.

The first published work describing a strong Raman signal from an interface was written by Fleischmann et al. in 1974. Fleischmann et al. reported that the Raman spectra of pyridine adsorbed on the roughened surface of silver had an unexpectedly high intensity [64,66]. The authors reported that the high intensity of the signal was the result of a large number of adsorption spots on the electro-roughened surface [67]. In 1977 teams led by Jeanmaire and van Duyne [24] and Albrecht and Creighton [18] independently proved that the high intensity of the Raman signal results from a large increase in the cross-section for Raman scattering of adsorbed molecules, known as Raman spectroscopy with surface enhancement. However, additional studies have shown that SERS is the result of a combination of two effects: the electromagnetic effect and the resonant charge transfer effect [24].

In recent years, the development of apparatus used for the detection of Raman spectra has increased interest in the SERS method. Besides, improvements in the field of nanotechnology have opened up new opportunities, particularly in the design and production of SERS substrate. As a result, the SERS technology is used in many areas, i.e., it plays an important role in improving the sensitivity and selectivity of the bioanalysis technique [68,69]. Biological research usually utilizes colloids of plasmonic nanoparticles as substrates. This is largely because gold and silver nanoparticles are easy to obtain by reducing the corresponding Au or Ag salts, and besides this nanoparticles can be applied to virtually any surface and SERS analyses can be performed [70]. Due to the presence of gold and silver colloids, Zeiri 2007 performed SERS measurements of biological samples for which the Raman spectra of the material itself are masked by strong fluorescence. The research material consisted of alfalfa seeds, green tea leaves, carrot root, and red cabbage leaves. Strong SERS spectra at 633 and 785 nm were measured for all samples tested with the use of gold colloidal solutions. Silver colloids did not create optimal conditions in this study. The measured spectra were weaker and showed more noise than the measurements with gold colloids. However, scientists failed to completely eliminate fluorescence. For shorter excitation wavelengths, fluorescence masked Raman spectra even in the presence of colloids [71]. Other results were obtained by Palanco 2015 in a study on the use of SERS spectroscopy in the analysis of plant material in onion with the use of silver colloids. The scientist obtained a strong Raman spectrum of chemical components such as cellulose, proteins, flavonols in the outer layer of onion. Moreover, owing to the use of silver colloids, he was able to study the deeper layers of the material. He pointed to the complex, heterogeneous chemical structure of plant tissues [72].

In 2018 colloids of nanoparticles were used by Kołątaj et al. in the analysis of the detection of thiuram pesticide on the surface of tomato peel. During the analysis, a bipyramidal solution of gold nanoparticles, additionally covered with a layer of silica, was applied to the peel of a tomato contaminated with pesticide. The Raman signal of the pesticide was measured on a surface prepared in this way [70]. The use of plasmonic nanoparticles allows for SERS measurements in liquids as well as in situ [73]. Surface-enhanced Raman spectroscopy is one of the most promising and safe techniques for direct detection of pathogens. SERS can be used to quickly identify harmful pathogens associated with alimentary intoxication, water pollution, and biological warfare [59,69].

Still, a major limitation in the SERS method is the strong fluorescence of biological components, which masks the Raman spectrum even in the presence of colloids. In addition, this method does not provide a quantitative measurement of the entire system, but only the activated materials. Complications related to the process to reach the stray Rayleigh band detectors constitute a limitation for SERS [29]. The charge transfer between the substrate and the molecule plays an important role in the SERS measurement, so the enhancement effect is limited to the first adsorbate layer only. Furthermore, SERS is a near-field effect and can only be exhibited by free-carrier materials [74].

### 3.2. Confocal Raman Spectroscopy (CRM)

Confocal Raman microscopy is an improved Raman spectroscopy system that provides information not only on the chemical composition but also on the internal structure of the material. The capability of CRM to provide spatial imaging of the sample in the x, y, and z axes, using lateral resolution, enables it to analyze at the micrometer level (<0.5 μm) [75]. In confocal Raman spectrometry, the immersion oil lens method or the dry lens method can be used [76].

**Figure 4:**
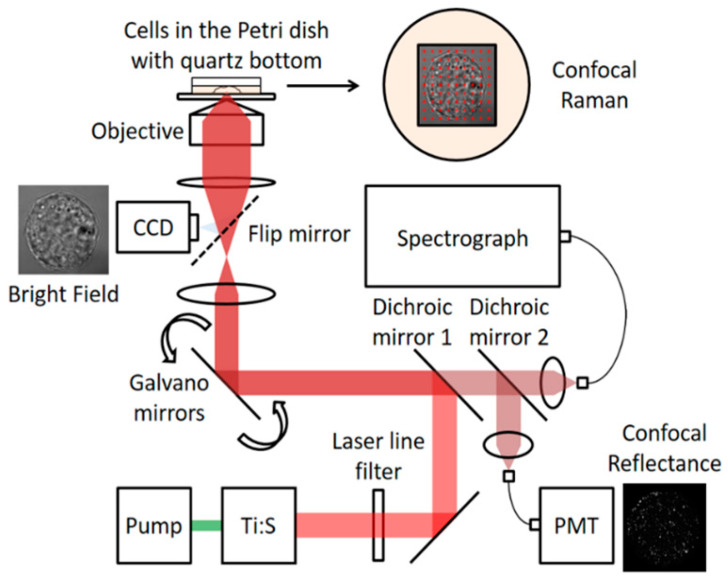
Depicts a general layout for confocal Raman microscopy.

The first confocal Raman microscope was invented in 1955 by Marvin Minsky [75]. In confocal Raman microscopy, the probe head works to focus the laser light on the sample through the microscope objective. Finally, the signal is collected on the camera of the charge-coupled device (detector) to generate the spectrum [18]. Figure 4 shows schematically the construction and operation of a confocal Raman spectrometer.

Due to its non-destructive nature, confocal Raman microscopy demonstrates great potential for in situ chemical analysis of plants [8,50,51,75]. CRM does not require extensive pre-treatment of the material or staining of the cell walls. It can provide an optical section of tissues without mechanical cutting or physical dissection [18,77]. CRM provides information on the chemical composition and distribution of individual substances in a non-invasive and non-labelling manner. Moreover, CRM allows structural information, i.e., crystallinity [78,79,80], to be obtained and also the depth profile of samples to be analyzed [80,81]. Depth profiling by confocal Raman microscopy can be used in two ways. The first method is to plot the intensity of the band selected as a function of the distance from the sample surface. This way it is possible to get information on the composition and the gradient-structure of the sample. The second potential way is to acquire the spectrum of deep structures and use it for identification [77]. CRM can also be used to directly visualize the variability of the polymer composition of the cell wall. The enormous advantage of confocal Raman microscopy is the ability to obtain full information about the spatial distribution of chemical components in the form of a hyperspectral image from a single measurement. When analyzing plant cells, all polysaccharides are examined at the same time [75]. The inelastic Raman scattering recorded as energy displacement by the CCD camera reflects the molecular vibrations of the material (e.g., stretching of bonds, rotation, twisting) and thus reflects its nature [82,83]. The Raman image consists of a thousand spectra in which each local position contains its own chemical information, and each spectral position has its own molecular identity [82,84]. The large amount of data generated by Raman imaging can be avoided by using multivariate methods that help in data management and interpretation [82]. Many authors have used a confocal Raman spectrometer to study layered systems to obtain detailed information on molecular composition [18,77]. In 2009, Zhang et al. in their experiment compared different CRM methods. Scientists found their advantages and disadvantages. The CRM lateral scanning through the cross-section yielded an excellent resolution but required destruction of the sample. The use of an immersion oil objective could improve the depth resolution, but the coating surfaces were easily contaminated by the immersion oil. The dry method required no sample preparation and was totally non-destructive; however, the relatively low depth resolution may be a disadvantage [76].

**Figure 5:**
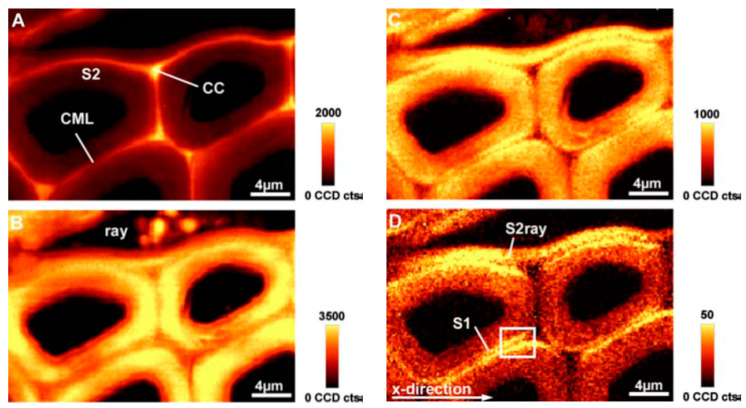
Raman confocal microscopy images of a cross section of poplar latewood. Raman bands: (**A**) 1550–1640 cm^−1^ (intensity of the aromatic lignin band); (**B**) 2780–3060 cm^−1^; (**C**) 1026–1195 cm^−1^; (**D**) 1090–1105 cm^−1^.

CRM is readily used in the case of biological tissues because of optical cutting to avoid the need to prepare any samples and to provide images of high spatial resolution [18,77]. Raman imaging techniques are successfully used to observe differences between wood tissues (example below Figure 5) or to monitor changes in the distribution of active substances in plant cells [51,52,85,86,87,88,89].

## 4. Raman Imaging of Plant Cell Walls

The non-destructive nature of Raman spectroscopy makes it extremely useful in the analysis of highly complex plant tissues [57]. To meet the requirements for a living plant cell walls must differ widely with regard to their composition and structure. Plant cell walls are nanocomposites of cellulose microfibers embedded in a matrix of poly-saccharides and aromatic polymers [90]. During the growth and maturation of plants, these very complex structures undergo changes and continuous modifications by changing the form of their cells and their composition [91,92]. The cell walls are responsible for the physical strength and shape of the cell [91]. Getting to know the plant’s morphology, composition, chemical constitution, and structure is extremely interesting. This knowledge enables scientists to answer the research questions of numerous fields including plant physiology, horticulture, and agriculture [57]

Raman techniques are an accurate tool for investigation into the chemical and structural properties of the plant tissues in a non-destructive manner, at micro-scale. The use of Raman spectroscopy permits the simultaneous tracking of all the polymers that are the building blocks of plant cell walls, providing information about the molecular structure of the polymers, i.e., crystallinity, spatial arrangement, and even deformation [90].

Raman microspectroscopy allows spectral maps without markers of tissues and biological cells to be obtained for chemical, structural, and environmental analysis. Raman images are spatially separated information about molecular structure, composition, and interactions on a micrometer or even nanometer scale, showing structural information that is important in the case of plant tissues [43,46,51,52,57]. An important advantage of the most modern experiments using Raman techniques at microscopic level is the possibility of using signal processing and multi-dimensional tissues to obtain an automatic analysis of the number of samples or plant fragments of one type [57].

Raman spectroscopy, with its various special techniques and methods, has been used for the study of plant biomass for nearly 30 years. The applications of Raman spectroscopy in plants are very extensive, ranging from research into structural polymers through metabolites to minerals. In the field of plant science, Raman was first used by Atalla and Agarwal to study tracheal cells in the xylem of woody tissues in the 1980s [93,94].

Technical developments in recent years have made Raman spectroscopy, together with its instrumentation, a precise tool for the study of plant tissues [94,95]. Raman microscopy is widely used to study plant cell wall composition [75,96], including xylem cells in *Arabidopsis thaliana* [82], *Cucumis sativus* [43], *Malus* sp. [97], *Populus* sp. [98,99], *Picea* sp. [17,50], and *Pinus* sp. [17]. Raman imaging techniques have been successfully used to observe the differences between various woody tissues and to monitor changes occurring in plant cells [88]. The multicomponent nature of the wood gives a vibration spectrum composed of widely overlapping bands. Scientists reported spectra of pure model components in order to detect their contributions in the Raman spectra of lignocellulosic substances. In 1997 Agarwal and Ralph noted that cellulose and lignin provide the most prominent Raman bands, while hemicelluloses and pectins have remained undetected due to their low content, broad Raman bands and overlapping with stronger bands of other components. In addition, researchers noted that cellulose and hemicelluloses have similar chemical bonds and therefore their bands are difficult to distinguish [100]. In 1980 Atalla proved that the Raman spectrum of cellulose has been shown to change in different orientations [101]. Similarly, in the study of poplar wood tissue, Gierlinger and Schwaninger 2006 obtained sharp bands of cellulose, which were influenced by orientation and crystallinity. They noticed that changes in cellulose orientation should be taken into account when tracking spatial changes in the intensity of cellulose bands [51]. In 1985 Atalla and Agarwal used confocal spectroscopy to study the secondary wall of earlywood tissue from Picea mariana (black spruce). Scientists have identified the spectrum of lignin shows small changes [49]. Other results were obtained by Gierlinger and Schwaninger 2006, who used the CRM method to illustrate changes in the molecular composition of the tissue of the secondary cell wall of poplar wood (*Populus nigra x Populus deltoids*). They visualized the spatial variability of lignin content and characterized the variable orientation of cellulose. In their imaging studies, unlike Atalla and Agarwal, no orientation effects were observed for lignin. Scientists say this is because small changes in lignin orientation are lost in much larger variations in lignin intensity imaged between different layers of the sample [51]. In 2006, Agarwal successfully applied confocal Raman microscopy to represent the complex organization of the wood cell wall. Scientists analyzed the distribution of lignin and cellulose in black spruce wood (*Picea mariana*). The obtained Raman images showed different concentrations of both lignin and cellulose between different morphological regions. The concentration of lignin was highest in the cell corner (CC), lignin concentration in compound middle lamella (CmL) was not different significantly in the secondary wall (S2 and S2–S3). In contrast, the degradation of cellulose showed the opposite pattern—low concentration in CC and CmL and high in S2 regions [50]. Hanninen et al. 2011 described the distribution of cellulose and lignin and quantity relative to alcohol and aldehyde groups in comparison with the total content of lignin in pine (*Pinus sylvestris*) and spruce (*Picea abies*) samples. The scientists found no significant differences in the breakdown of lignin and cellulose between these samples. Significant differences between the samples were observed in the decomposition of coniferyl alcohols and coniferyl aldehyde. Scientists have shown that the lignin/cellulose ratio is largely similar in the wood species studied, but the distribution of lignin may already vary considerably [17].

Using Raman’s instrumentation, in 2000 Manfait et al. investigated the presence of starch and proteins as well as the composition of cell walls in wheat grain [102]. In 2010 Lopez-Sanchez et al. described a study in which they investigated the option of using Raman spectroscopy to assess changes occurring during the development and ripening of olive fruit. They measured the spectra of different parts of the olive (peel, flesh, and stone) at different stages of development. Scientists noted an increase in the content of carotenoids and phenolic compounds in the growth phase of olives and their decrease during the ripening phase. They proved that Raman spectrometry is a suitable technique to monitor the content of carotenoids and phenolic compounds in olive fruit [103]. In 2011 and 2012 Qin et al. used the Raman spectrometry technique to visualize differences in ripening fruits. Raman spectrometry was used to visualize changes in carotenoid content at various stages during the maturing of tomato fruit. Researchers initially investigated the mechanism of carotenoid formation during tomato ripening using chemical Raman images of cut tomatoes. The uneven production of carotenoids during ripening gave researchers the opportunity to non-destructively assess the inner ripeness of tomatoes [86,104]. In 2014 Cabrales and his team used CRM to investigate the cross sections of cotton fibers harvested at different stages of growth. Researchers analyzed the Raman bands assigned to cellulose. The results of this study indicated that CRM can provide useful information on the deposition of cellulose during the development of cotton fibers [80]. With the help of CRM, Chylińska et al., 2014, investigated the distribution of pectins and cellulose polysaccharides in the cell wall of minimally prepared samples of tomato pulp [89]. In 2017 Chylińska and her team using a confocal Raman Spectroscopy discovered an impact of biochemical parameters on cell wall structure during physiological tomato fruit development. This study showed the chemical images for external factors in spatial distribution of polysaccharides in plant cell wall. This method allows to remark cell wall degradation during tomato fruit ripening (mainly pectic polysaccharides degradation) [105]. In 2016, Szymańska-Chargot et al. recorded the distribution of polysaccharides in the cell walls of apple flesh using confocal Raman microscopy. The paper describes changes in the content of polysaccharides in the cell wall, their structure and distribution during the ripening of apples on the tree and during storage. Raman images of apple cell walls emphasize significant changes in the number and location of major cell wall polysaccharides. In the period of apple ripening and ageing, changes in the distribution of pectin were demonstrated going from a layout dispersed along the cell wall in young fruits to concentrated in the corners of the cell walls in ripe and stored fruits [88]. Heiner et al., 2017, in research on the chemical structural properties of the sorghum root and leaf tissue, combined the spontaneous Raman phenomenon with two-photon fluorescence and the second harmonic generation phenomenon. They showed that the combination of these different microspectroscopies provides comprehensive information on the histology, molecular structure, and cellular composition of plant tissues [57]. Zeise et al., 2018, analyzed Raman images of sections from various tissues of *Cucumis sativus* plants. When imaging individual tissue snips, researchers obtained detailed histological information, including the size and number of cells, and the thickness of the cell walls [43]. Mateu et al., 2018, analyzed the cell wall components in spruce wood micro-sections as well as in *Arabidopsis thaliana* stems using confocal Raman spectroscopy. Researchers were able to isolate different layers of cell walls [90]. Dinat et al., 2018, using a Raman spectrometer, analyzed the differences in the phloem and xylem spectra from sections of *Arabidopsis thaliana* flower stems for both the wild and mutant strains [106]. In 2018 Zhu, Wu, and Chen established a method using the Raman microspectroscopy technique to visualization loquat fruit lignification at the cell level. This method can be used in the future to track changes in texture over the ripening and post-harvest storage process of different fruits and vegetables [107]. Pecinar et al., 2019, investigated the occurrence of possible differences in carotenoids in several fruit species using Raman spectroscopy, i.e., rose, nectarine, plum, pepper, corn, and tomato. The team noticed changes in carotenoids, especially in the pericarp and its section, during the ripening of tomato fruit [94]. Wykręt and Dulski 2019, using a Raman spectrometer, analyzed the main polymers of the cell wall of non-fibrous plants and the distribution of selected polymers in the cell wall in its native state [108]. He, Zabotina, and Yu 2020 using the confocal Raman Microscopy revealed the polysccharide organization and distribution inside the onion plant cell wall. Researchers explored the interaction between the cell wall polysaccharides and pectin enzyme endo-polygalacturonase (EPG). Analysis of Raman spectral revealed the pectin distribution and pectin interaction with the EPG. On the basis of Raman images, authors deduced information on the coexistence and colocalization of pectin, hemicellulose, and cellulose [109].

## 5. Summary

Simplicity in using and collecting data, as well as conducting analyses without the need for prior labelling and complicated sample preparation, has resulted in increased interest and a significant increase in the use of Raman spectroscopy in the field of life sciences. Raman spectroscopy techniques used for research on biological material, i.e., plant tissues, provide insight into the structure and organization of the plant cell wall. Raman microscopy is a precise tool for the study of the structural polymers, metabolites, and mineral substances of plant cells and tissues. Owing to its exceptional sensitivity, this method can distinguish even subtle differences among regions with different chemical composition and structure. It allows the researcher to study the processes taking place in the plant tissues and cells.

The introduction of new and improved Raman techniques by researchers, as well as constant technological development of the apparatus, has resulted in an increased significance of Raman spectroscopy in the discovery and definition of tissues and the processes taking place inside them. The Raman technique is a promising direction for science resulting in the discovery of plant tissues as well as their identification and classification. This method offers extensive potential for use in further scientific work on complex plant material.

In plants, many factors can influence the proper growth, health, and disease resistance [110]. In the future, special attention should be paid to the use of Raman techniques for carrying out the research on the impact of various factors, such as environmental changes, environmental stress on biological processes, and the general condition of plants. It is very important to monitor plants and detect individual lesions at a very early stage, before any externally visible signs appear. Raman spectroscopy is a suitable technique for this [110]. Scientists can measure plant responses to external factors ex vivo and in vivo. The research results obtained with the help of Raman spectroscopy in conjunction with spectral databases will allow us to keep track of the processes taking place during plant growth, simultaneously in all its parts. This method can provide a basis for recognizing and describing plant responses to adverse factors. The collected fingerprints can be interpreted and related to the health of the plant and the factor involved. By comparing the studied spectrum with the spectrum library, one can find the reason for the changes taking place at a very early stage. Concentration on research that will bring closer the recognition of complex plant cell responses to stress factors and environmental changes will bring definite benefits in the cultivation of fruit and vegetables on a global scale.

## Data Availability

Not applicable.

## Abbreviations

AFM	Atomic force microscopy
Ag	Silver
Au	Gold
CARS	Coherent anti-Stokes Raman scattering
CRM	Confocal Raman Spectroscopy
EMF	electromagnetic field
FT	Fourier transform
GAGs	glycosaminoglycans
LSP	Local surface plasmon
NIR	Near Infrared Spectroscopy
RRS	Raman resonance scattering
SERS	Surface-enhanced Raman scattering
SPM	Scanning probe microscopy
SRS	Stimulated Raman scattering
STM	Scanning tunneling microscopy
TERS	Tip-enhanced Raman
UV-VIS	Ultraviolet/Visible Spectroscopy
